# The impact of brain lesions on health-related quality of life in patients with WHO CNS grade 3 or 4 glioma: a lesion-function and resting-state fMRI analysis

**DOI:** 10.1007/s11060-023-04254-1

**Published:** 2023-02-07

**Authors:** Alexander Heinzel, Felix M. Mottaghy, Christian Filss, Gabriele Stoffels, Philipp Lohmann, Michel Friedrich, Nadim J. Shah, Svenja Caspers, Carolin Weiss Lucas, Maximilian I. Ruge, Norbert Galldiks, Gereon R. Fink, Karl-Josef Langen, Martin Kocher

**Affiliations:** 1 Institute of Neuroscience and Medicine, Research Center Juelich, INM-1, -3, -4, -11, Juelich, Germany; 2 Department of Nuclear Medicine, RWTH Aachen University Hospital, Aachen, Germany; 3 Department of Nuclear Medicine, Martin-Luther-University Halle-Wittenberg, Halle, Germany; 4 Department of Radiology and Nuclear Medicine, Maastricht University Medical Center, Maastricht, Netherlands; 5 Center for Integrated Oncology (CIO), Universities of Aachen, Bonn, Cologne, and Duesseldorf, Germany; 6 Juelich-Aachen Research Alliance (JARA), Section JARA-Brain, Juelich, Germany; 7 Department of Neurology, RWTH Aachen University Hospital, Aachen, Germany; 8 Institute for Anatomy I, Medical Faculty and, University Hospital Duesseldorf, Heinrich Heine University Duesseldorf, Duesseldorf, Germany; 9 Department of General Neurosurgery, Faculty of Medicine and, University Hospital Cologne, University of Cologne, Cologne, Germany; 10 Department of Stereotaxy and Functional Neurosurgery, Center for Neurosurgery, Faculty of Medicine and, University Hospital Cologne, Cologne, Germany; 11 Department of Neurology, Faculty of Medicine and, University Hospital Cologne, University of Cologne, Cologne, Germany

**Keywords:** Glioma, Quality of life, Brain networks, Functional magnetic resonance tomography, Positron emission tomography, Radiotherapy

## Abstract

**Purpose:**

In glioma patients, tumor development and multimodality therapy are associated with changes in health-related quality of life (HRQoL). It is largely unknown how different types and locations of tumor- and treatment-related brain lesions, as well as their relationship to white matter tracts and functional brain networks, affect HRQoL.

**Methods:**

In 121 patients with pretreated gliomas of WHO CNS grades 3 or 4, structural MRI, O-(2-[^18^F]fluoroethyl)-L-tyrosine (FET) PET, resting-state functional MRI (rs-fMRI) and self-reported HRQoL questionnaires (EORTC QLQ-C30/BN20) were obtained. Resection cavities, T1-enhancing lesions, T2/FLAIR hyperintensities, and lesions with pathologically increased FET uptake were delineated. Effects of tumor lateralization, involvement of white matter tracts or resting-state network nodes by different types of lesions and within-network rs-fMRI connectivity were analyzed in terms of their interaction with HRQoL scores.

**Results:**

Right hemisphere gliomas were associated with significantly less favorable outcomes in physical, role, emotional and social functioning, compared with left-sided tumors. Most functional HRQoL scores correlated significantly with right-sided white-matter tracts involvement by T2/FLAIR hyperintensities and with loss of within-network functional connectivity of right-sided nodes. Tumors of the left hemisphere caused significantly more communication deficits.

**Conclusion:**

In pretreated high-grade gliomas, right hemisphere lesions are associated with reduced HRQoL scores in most functional domains except communication ability, compared to tumors of the left hemisphere. These relationships are mainly observed for T2/FLAIR lesions involving structural and functional networks in the right hemisphere. The data suggest that sparing the right hemisphere from treatment-related tissue damage may improve HRQoL in glioma patients.

**Supplementary Information:**

The online version contains supplementary material available at 10.1007/s11060-023-04254-1.

## Introduction

Gliomas of the WHO central nervous system (CNS) grades 3 or 4 are the most common primary brain tumors in adults. They are characterized by rapid and infiltrative growth resulting in a mostly unfavorable prognosis despite therapeutic efforts [1, 2]. Patients suffer not only from brain damage due to destructive tumor growth but also from possible side effects of various therapies such as tumor resection, radiation, chemotherapy, and their combinations, which affect the patients' functioning and well-being. [3, 4]. As a result, long-term survivors endure a significant symptom burden leading to a reduced health-related quality of life (HRQoL) [5, 6]. In these patients, HRQoL can therefore be considered as important as the survival time. Consequently, the assessment of patients’ quality of life has become an important aspect in the evaluation of treatment strategies in addition to well-established outcome parameters such as overall survival, progression-free survival, or imaging response [7].

One of the most commonly used instruments to evaluate HRQoL in clinical trials related to brain tumors is the European Organization for Research and Treatment of Cancer (EORTC) Quality of Life Questionnaire Core 30 (QLQ-C30) [8] with its accompanying brain-specific module BN20 [9]. The QLQ-C30 is a 30-item self-report questionnaire that, apart from a selection of symptoms commonly reported by cancer patients, assesses performance in five functional domains (physical, role, emotional, cognitive and social functioning), and a score for overall or global quality-of-life (global QL). The BN20 questionnaire comprises 20 items, most of which are also grouped into four functional scales (future uncertainty, visual disorder, motor dysfunction, communication deficit), and seven single symptom scores. Thus, this instrument is based on a multidimensional concept of health-related HRQoL comprising physical, mental, emotional, and social domains. Currently, it is unclear how these HRQoL ranges relate to tumor location or to regional or network brain damage. Such knowledge is critical for clinical decision-making and provides valuable information on the relationship between brain lesion location and dysfunction [10].

So far, studies of higher brain functions in glioma patients have mainly focused on cognitive performance. In a longitudinal study on patients with high-grade gliomas, Dallabona et al. [11] found that, among other factors, tumor lateralization and overall mass effect can contribute to the prediction of postoperative cognitive performance. Patients with major deficits in language and verbal memory tasks predominately suffered from left lateralized tumors. These findings align with data from Habets et al. [10] who investigated the impact of tumor location on neurocognitive functioning (including six cognitive domains) by applying MRI-based lesion-symptom mapping. They suggested that deficits in the different cognitive domains might be related predominantly to the left hemisphere with patients suffering from left hemispheric gliomas showing the highest likelihood of cognitive deficits. Furthermore, a recent study showed that multimodal imaging can predict cognitive deficits in glioma patients [12]. T2/FLAIR abnormalities correlated best with cognitive outcomes. Moreover, most functional cortical nodes and white matter tracts related to cognitive performance were left-hemispheric. In particular, deficits in language-related functions could be linked to T2/FLAIR hyperintensities in the nodes and tracts of the left temporal lobe.

However, it remains unclear how these findings relate to the patients’ HRQoL since this construct comprises additional domains such as emotional and social functioning as well as aspects of physical well-being. Emotional processing has been notably related to the right hemisphere. The so-called “right hemisphere hypothesis” posits a general dominance for emotional processing irrespective of the type of emotional valence [13–16]. Based on this theory, we hypothesized involvement of the right hemisphere in the neural correlates of HRQoL in pretreated high-grade glioma patients. Therefore, we analyzed the relationship between location and extent of MRI-assessed resection cavities, T1-enhancing lesions, T2/FLAIR hyperintensities, and regions with pathologically increased O-(2-[^18^F]fluoroethyl)-L-tyrosine (FET) uptake and the scores for functional domains assessed from the EORTC questionnaires QLQ-C30 and BN20 that are potentially associated with higher, integrative brain function. Moreover, we assessed the correlation of within-network node connectivity of seven major functional magnetic resonance imaging (fMRI) resting-state networks with the HRQoL scores.

## Patients and methods

### Study population

The study population was identical to that investigated in a previous study undertaken by our group [12]. Briefly, it originated from a prospective, cross-sectional study and comprised 121 patients (February 2018 to September 2020) suffering from WHO CNS grade 3 or 4 gliomas, according to the 2016 WHO classification [17]. Patients were referred for hybrid MR/PET imaging for assessment of residual tumor after surgery, therapy monitoring or suspicion of recurrent tumor after completion of primary therapy and thus represent a cross-section through different stages of the course of disease, including some patients who had not fully completed primary treatment.

The inclusion criteria were: ECOG performance score at screening of 0–1, no major depression, no seizures, and fluency in the German language. The local ethics committee approved the protocol, and all patients provided informed written consent according to the Declaration of Helsinki. Patient characteristics including the symptoms present at imaging and the main lobe involved are shown in Table 1. The median interval between therapy initiation and imaging was 14 months (mean, 30 months; range, 1–214 months), and 105 patients had completed tri-modality primary treatment, including biopsy/resection, definitive/postoperative radiotherapy, and concomitant/adjuvant chemotherapy with alkylating agents. Most patients had already received one series (100 patients) or two series (14 patients) of local radiotherapy (dose range for first series 59–62 Gy in 92% of patients, usually applied within 6 weeks), with a median interval of 13 months (mean, 32 months; range, 2–213 months) between the start of irradiation and imaging. All patients except one were right-handed.

**Table 1:** Patient characteristics

	All Glioma Patients (n = 121)	Left-Sided Glioma (n = 65)	Right-Sided Glioma (n = 56)	p
Gender (male/female)	73/48	36/29	37/19	0.23
Age (years)	52 (28–74)	52 (28–74)	52 (29–73)	0.88
ECOG-PS (0/1–2)	58/63	34/31	24/32	0.30
Education (ISCED-Score)	8 (3–10)	8 (3–10)	7 (3–10)	0.58
Employment (no/yes)	45/76	23/42	22/34	0.40
Follow-up interval (months)	14.4 (0.6–213.7)	14.7 (0.6–190.9)	13.5 (0.6–213.7)	0.69
Presenting symptoms				
Aphasia	17	15	2	*0.002*
Paresis	29	12	17	0.13
Fatigue	19	8	11	0.27
Vision	12	5	7	0.38
Vertigo, confusion	4	2	2	0.88
Tumor type				
GBM, IDH wildtype	67	31	36	0.49
GBM, IDH mutant	10	7	3	–
GBM, NOS	4	3	1	–
AA, IDH wildtype	5	2	3	–
AA, IDH mutant	16	9	7	–
AA, NOS	7	5	2	–
AOD, IDH-mut&1p19q codel	12	8	4	–
Tumor location*				
Frontal	59	31	28	0.90
Parietal	15	7	8	–
Temporal	38	22	16	–
Occipital	9	5	4	–
Lesion volumes				–
Resection cavity (mL)	9.3 (0–172.5)	9.0 (0–161.5)	9.6 (0–172.5)	0.88
FLAIR hyperintense (mL)	53.4 (3.4–252.9)	48.7 (3.4–252.9)	56.2 (4.4–232.6)	0.062
T1 contrast enhancing (mL)	5.4 (0–122.9)	2.5 (0–88.0)	8.1 (0–122.9)	0.13
FET-PET pathol. Uptake (mL)	14.9 (0–227.8)	8.2 (0–127.3)	20.1 (0–227.8)	0.066
Treatment (Number of Procedures)				
Surgery# (1/2/3/4)	101/17/2/1	54/9/1/1	47/8/1/0	0.83
Radiotherapy (0/1/2)	7/100/14	4/55/6	3/45/8	0.43
Chemotherapy (0/1/2/3)	10/91/16/4	8/48/5/4	2/43/11/0	0.15
Corticosteroids (no/yes)	91/30	51/14	40/16	0.25
Anticonvulsants (no/yes)	49/72	27/38	22/34	0.47
FET pathol. Uptake (no/yes)	63/58	36/29	27/29	0.27

Median (Range) unless otherwise stated; *ECOG*-*PS* eastern cooperative oncology group performance score; *ISCED* international standard classification of education (1997); *GBM* glioblastoma; *NOS* not otherwise specified; *AA* anaplastic astrocytoma; *AOD* anaplastic oligodendroglioma; *IDH* Isocitrate-Dehydrogenase; 1p19q *codel* 1p19q codeleted; *FET* O-(2-[18F]fluoroethyl)-L-tyrosine; *main lobe involved #including biopsy

### Assessment of quality of life

Quality of life was assessed at the date of imaging using the validated German version of the EORTC Quality of Life Questionnaire QLQ-C30 and the EORTC QLQ-BN20 (https://qol.eortc.org) [8, 18]. The QLQ-C30 assesses five functional domains (physical, role, emotional, cognitive, social), an overall or global quality-of-life (global QL) scale, three symptom scales (pain, fatigue, nausea/vomiting) and six single items (dyspnea, insomnia, anorexia, diarrhea, constipation, financial difficulties). The BN20 questionnaire comprises 20 items most of which are grouped into four functional scales (future uncertainty, visual disorder, motor dysfunction, communication deficit), and seven single symptom scores (headaches, seizures, drowsiness, hair loss, itchy skin, weakness of legs, bladder control). The final scores for the scales and items were computed according to the EORTC QLQ scoring manuals, where the raw scores from one question or the average score from 2 to 5 questions are rescaled to a range of 0–100 points. Additionally, sociodemographic variables comprising age, gender, and education (International Standard Classification of Education, ISCED, http://uis.unesco.org/sites/default/files/documents/ international-standard-classification-of-education-1997-en_0.pdf) were recorded.

### Multimodal imaging, image segmentation, and determination of lesion-specific damage to functional cortical regions and white matter tracts

These procedures have been described in detail before [12]. Briefly, FET positron emission tomography (PET) and MR data were acquired simultaneously using a high-resolution 3 T hybrid PET/MR scanner (Siemens Tim-TRIO/BrainPET, Siemens Medical Systems, Erlangen). Structural MR imaging included a 3D T1-weighted magnetization-prepared rapid acquisition gradient-echo (MPRAGE) anatomical scan, a contrast-enhanced T1-weighted image (T1-CE) obtained from a second MPRAGE scan following the injection of gadolinium, or high-resolution T1-weighted, contrast-enhanced MR scans available from the referring institution. Moreover, T2-weighted and T2-weighted fluid-attenuated inversion recovery (FLAIR) structural images were acquired. In addition to these imaging procedures, all patients underwent resting-state fMRI (rs-fMRI), where 300 functional volumes were acquired within 11 min using a gradient-echo echo planar imaging (GE-EPI) pulse sequence (36 axial slices, slice thickness 3.1 mm, repetition time TR = 2200 ms, echo time TE = 30 ms, flip angle = 90°, FoV = 200 × 200 mm^2^, in-plane voxel-size 3.1 × 3.1 mm^2^). The patients were instructed to relax and to let their minds wander but not to fall asleep [12].

**Fig. 1:**
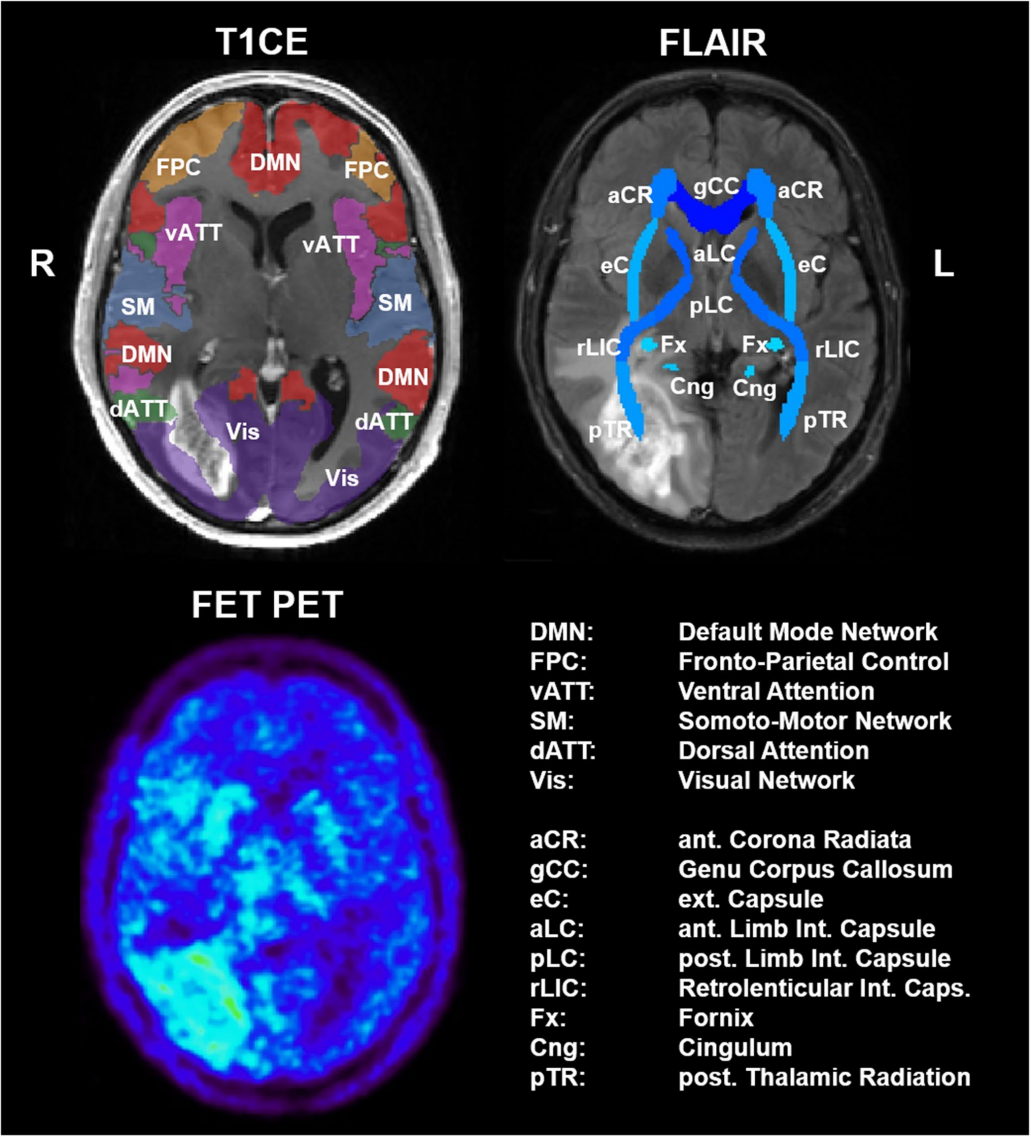
Principles of lesion mapping demonstrated in a patient with a right temporol-occipital glioma. Contrast enhancing tumors, T2/FLAIR hyperintensities, regions with pathologically increased FET PET uptake and resection cavities were overlaid on atlases of fMRI resting-state networks (2 × 50 nodes from 7 networks) and white matter tracts (2 × 24 tracts). All images and segments were normalized to the MNI-152 standard space.T1CE: T1 weighted contrast-enhanced; FLAIR: fluid-attenuated inversion recovery; FET PET: O-(2-[^18^F]fluoroethyl)-L-tyrosine positron emission tomography

Lesion masks were generated for resection cavities, T1-CE enhancing lesions, T2/FLAIR hyperintensities, and lesions with pathologically increased FET uptake. Resection cavities were manually contoured by a radiation oncologist (M.K.), whereas the T1-CE-enhancing lesions and T2/FLAIR hyperintensities were automatically segmented (deep-learning-based software HD-GLIO-AUTO, https://github.com/NeuroAI-HD/HDGLIO-AUTO). FET PET segmentation was implemented by an FSL (https://fsl.fmrib.ox.ac.uk) custom script using a tumor-to-brain ratio (TBR) of > 1.6 as the threshold for pathological FET uptake [19]. All segmentations were visually inspected and manually corrected if needed. For lesion-function mapping, the rs-fMRI-based cortical parcellation atlas of Schaefer et al. [20], comprising 2 × 50 functional nodes belonging to 7 resting-state networks (visual, somato-motor, dorsal attention, ventral attention, limbic, fronto-parietal control, and default mode), and an atlas of white matter tracts (2 × 24 tracts) from the Stereotaxic White Matter Atlas of the Johns Hopkins University (JHU) [21] were applied. All images were spatially normalized by elastic registration to the MNI-152 brain template by means of the SPM12 toolbox (www.fil.ion.ucl.ac.uk/spm/ software/spm12) and, finally, the partial overlapping volume of the nodes or tracts with each of the lesion segments was computed, see Fig. 1.

### Determination of resting-state functional connectivity

The determination of resting-state functional connectivity (RSFC) was performed similarly to Kocher et al. [22]. Briefly, functional images were subjected to the standard pre-processing steps of the SPM12/CONN toolbox comprising motion correction with removal of outliers and cerebro-spinal fluid/ white matter signals, slice timing correction, smoothing with 5 mm FWHM, bandpass-filtering to 0.008–0.09 Hz and denoising. All structural and functional images were non-rigidly co-registered to the MNI-152 standard brain template using the SPM12/CONN unified segmentation/registration algorithm. For the determination of RSFC, the cortical parcellation of Schaefer et al. [20] was imported into the CONN toolbox and used to compute full connectivity matrices from the z-transformed Pearson correlation coefficients between the respective nodes. From these z-values, the within-network connectivity for each node of the respective network [23] was computed.

### Statistical analysis

SPSS (IBM SPSS Statistics version 27, IBM Corporation, Armonk, NY, USA) and the Python software package scipy.stats (version 1.5.4, https://scipy.org) were used for all analyses. Correlation coefficients between EORTC QLQ domain scores and the volumetric overlap between lesions and functional nodes or white matter tracts were computed using a 2-sided Kendall tau-b rank correlation that corrects for ties and is less sensitive to outliers. Moreover, the Pearson correlation coefficients between within-network functional connectivity of individual nodes and the EORTC QLQ domain scores were calculated. No formal correction was applied for massive univariate testing [24]. However, only correlations with p < 0.001 for lesion-HRQoL and p < 0.01 for RSFC-HRQoL analysis were considered significant. Differences in patient characteristics and HRQoL scores between left- and right sided tumors were compared by appropriate application of two-sided t-tests, Mann–Whitney U-tests or Chi-Square tests where p-values < 0.05 were regarded significant.

In addition, effect sizes were computed using the definitions of Cohen [25]. The effect size for the comparison of HRQoL scores between left- and right-sided tumors was calculated from Cohen’s *d* by dividing the difference of the means through their pooled standard deviations, and was classified as representing small (≥ 0.2), medium (≥0.5) or large (≥0.8) effects. We also adopted the definition of minimal clinically important differences from Osoba et al. [26], where differences in HRQoL scores were rated as minimal (5–10 points on a 100-point scale), moderate (10–20 points) or very much (> 20points). As also suggested by Cohen, correlation coefficients of 0.1, 0.3 and 0.5 where assumed to represent small, medium or large effect sizes.

## Results

### EORTC health-related quality-of-life scores

**Fig. 2:**
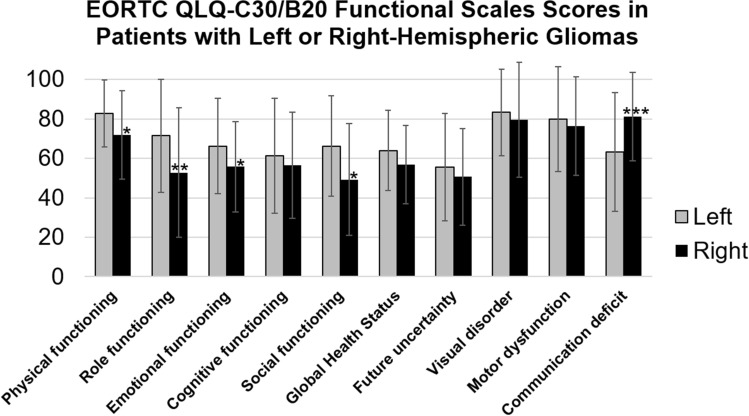
EORTC HRQoL functional scores in patients with left or right-sided gliomas. *p < 0.05, **p < 0.01, ***p < 0.001, Mann–Whitney U-test. For all domains, lower scores indicate lower performance. All significant differences were moderate (> 10 points) in terms of clinical importance

As shown in Fig. 2, patients with right-hemispheric gliomas had lower EORTC functional HRQoL scores in 9/10 domains and scored significantly lower for physical, role, emotional, and social functioning. Conversely, communication deficits were significantly more pronounced in patients with left-sided gliomas. Apart from emotional functioning (small effect size), all other significant differences had a medium effect size, and in all cases the difference was moderate (> 10 points) in terms of clinical importance [26]. Of note, except for the prevalence of (mild) aphasia, patients with left or right hemispheric gliomas showed no significant differences in disease-related, treatment-related, or sociodemographic characteristics (Table 1). Apart from a weak association of an increased age with a reduction in (self-reported) cognitive functioning (ρ = − 0.18, p = 0.045) and a correlation of higher educational status with a lowered future perspective (ρ = − 0.20, p = 0.027), the sociodemographic status did not correlate with the functional HRQoL measures. Regarding the symptom scales and scores, the patients with right-sided tumors suffered significantly more from fatigue, financial difficulties, itchy skin and weakness of legs (Fig. S1, supplement).

### Correlation of lesion volume and location with global health status and functional HRQoL scores

**Fig. 3:**
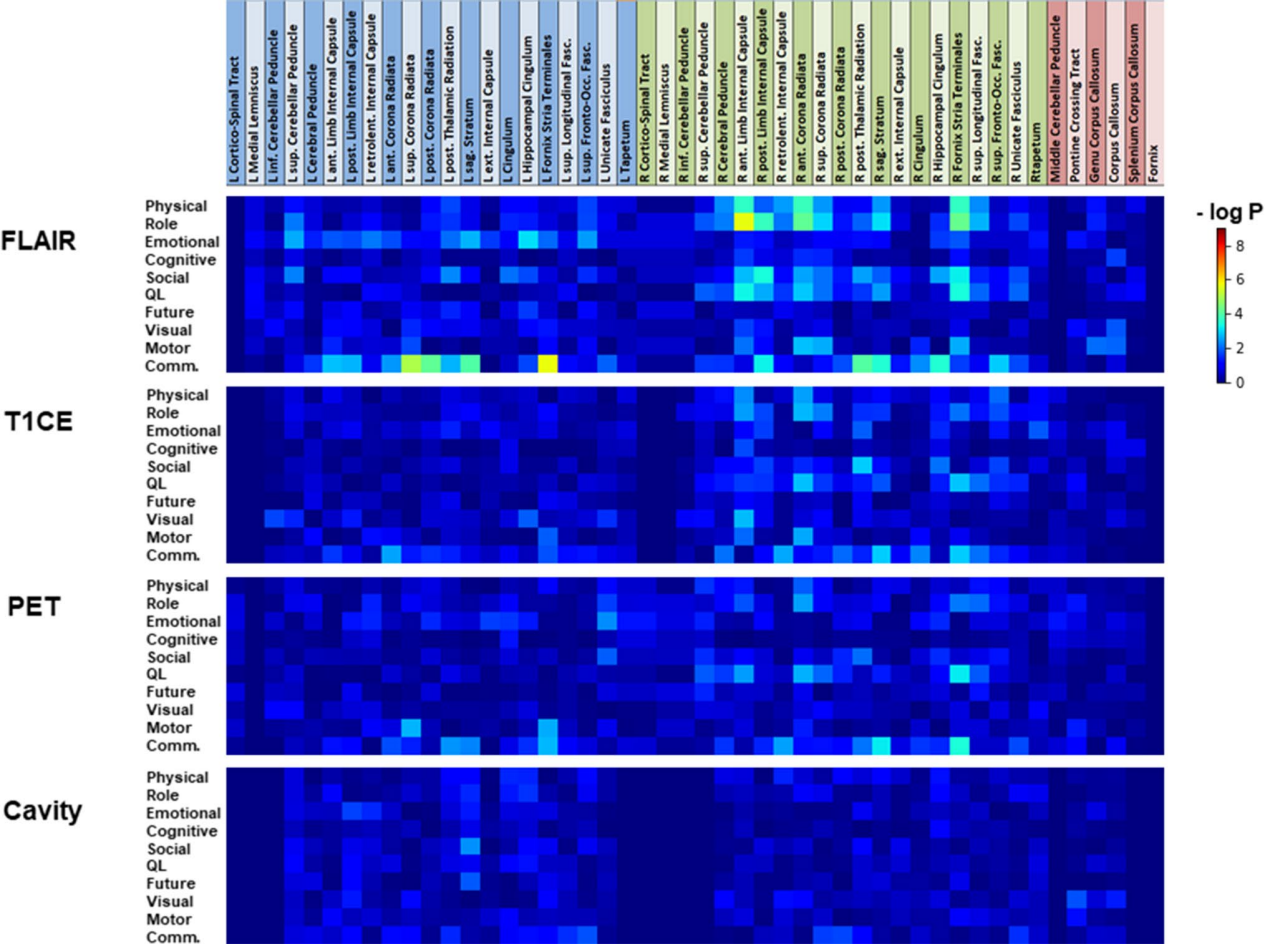
Heatmap for p-values of correlation analysis (Kendall tau) between partial volumetric affection of white-matter tracts and EORTC HRQoL scores in patients with high-grade gliomas. The top row shows the list of left-sided (blue), right-sided (green), and midline (red) tracts. Physical: physical functioning; Role: role functioning; Cognitive: cognitive functioning; Social: social functioning; Global QoL: global health status/ QoL; Future: future uncertainty; Visual: visual disorder; Motor: motor dysfunction; Comm.: communication deficit

**Fig. 4:**
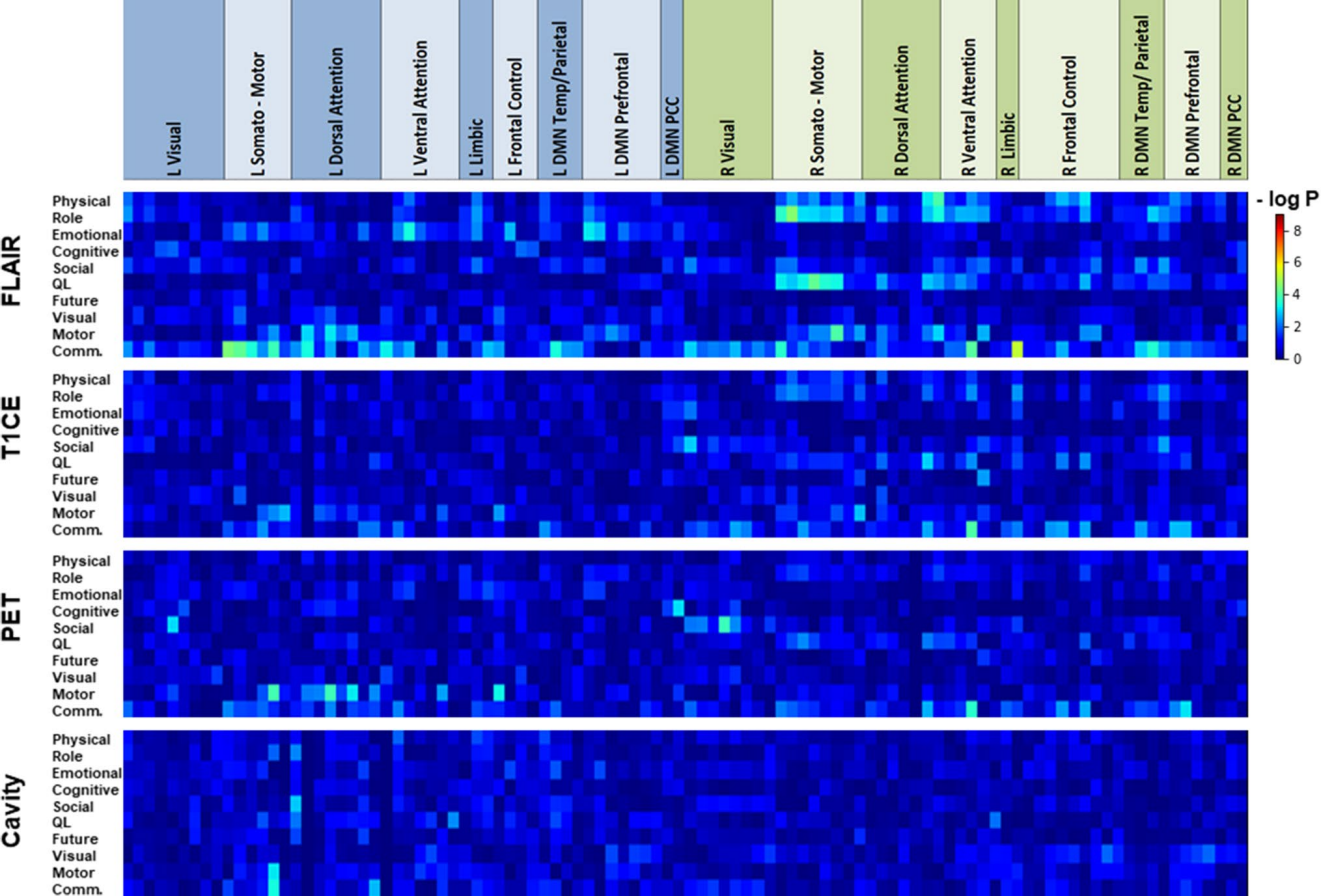
Heatmap for p-values of correlation analysis (Kendall tau) between volumetric affection of functional cortical areas and EORTC HRQoL scores in patients with high-grade gliomas. The top row indicates membership of the 2 × 50 nodes to the left-sided (blue) and right-sided (green) parts of 7 resting-state networks. DMN: default mode network; PCC: posterior cingulate cortex. Physical: physical functioning; Role: role functioning; Cognitive: cognitive functioning; Social: social functioning; Global QoL: global health status/ QoL; Future: future uncertainty; Visual: visual disorder; Motor: motor dysfunction; Comm.: communication deficit

Regarding the total volumes of each lesion type, only the global health status was inversely correlated with the volumes of the lesions (FLAIR hyperintensities: ρ = − 0.25, p = 0.05, T1-enhancing lesions: ρ = − 0.24, p = 0.009, pathological FET uptake: ρ = − 0.27, p = 0.003). The p-value maps for the correlation analysis between the partial volumetric involvement of tracts or nodes by the different kinds of lesions and the HRQoL measures are depicted in Fig. 3, 4.

As shown in Fig. 3, HRQoL scores mainly correlated (p < 0.001) with the involvement of white-matter tracts of the right hemisphere by T2/FLAIR hyperintensities. In particular, physical functioning, role functioning, social functioning, and global health status were associated with an involvement of the right anterior and posterior limbs of the internal capsule, the right anterior corona radiata, and the right striae terminales of the fornix. Conversely, communication deficits were more strongly associated with the left corona radiata and left striae terminales of the fornix. Most of the coefficients (17/22) for the significant correlations were negative in the range of − 0.24 to − 0.36, thus representing small to medium effect sizes and indicating a detrimental effect of tract involvement on different aspects of HRQoL (Fig. S2 suppl.). Other types of lesions showed only weaker correlations, which, however, still prevailed in the right hemisphere.

**Fig. 5:**
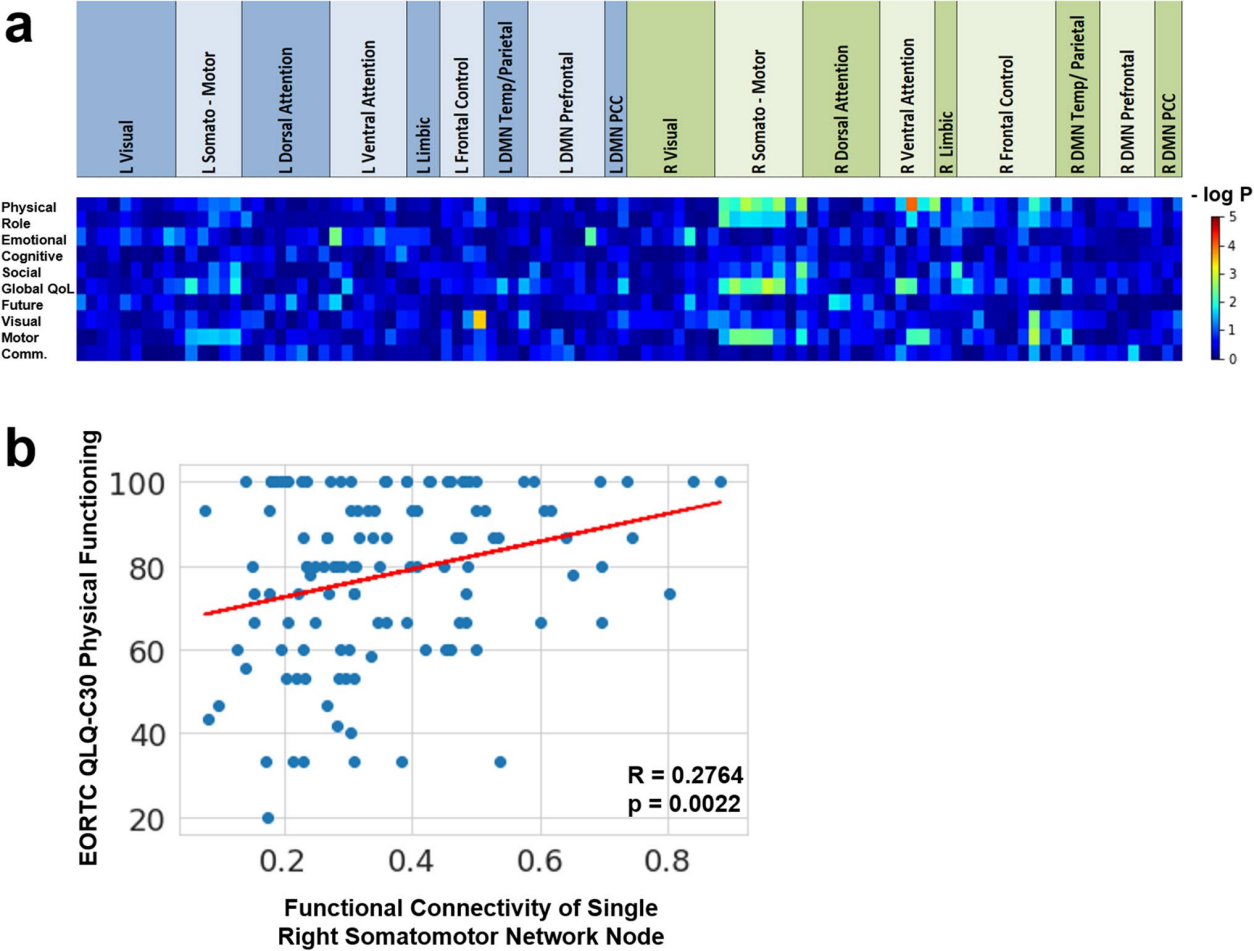
**a** Heatmap of p-values of Pearson correlation analysis between within-network functional connectivity of individual cortical nodes and EORTC HRQoL scores in patients with high-grade gliomas. The top row indicates the membership of the 2 × 50 nodes to the left-sided (blue) and right-sided (green) parts of 7 resting-state networks. DMN: default mode network; PCC: posterior cingulate cortex. Physical: physical functioning; Role: role functioning; Cognitive: cognitive functioning; Social: social functioning; Global QoL: global health status/ QoL; Future: future uncertainty; Visual: visual disorder; Motor: motor dysfunction; Comm.: communication deficit. **b** Correlation analysis between the within-network resting-state functional connectivity of a right-sided somatomotor node (#64) and the EORTC QLQ-C30 physical functioning score in patients with high-grade gliomas. R: Pearson correlation coefficient, p: p-value

The same principal correlation patterns and effect sizes were observed when analyzing the involvement of functional cortical nodes by FLAIR lesions (24/30 significant correlations with negative coefficients; Fig. S3, suppl.). Physical and role functioning and global health status were mainly associated with the involvement of the right somato-motor region and other nodes of the right hemisphere, while the severity of communication deficits correlated with several nodes of the left hemisphere. As shown in Fig. 5a and Fig. S4 (suppl.), the within-network functional connectivity of several nodes of the right hemisphere correlated positively (27/29 significant correlations) with the HRQoL scores. Reduced connectivity of most of the nodes of the right somato-motor network and the right ventral attention network was associated with a significant decline in physical, role, and motor function, as well as global health status. A representative example of the correlation between the functional connectivity of a right-hemispheric somato-motor node and the physical functioning score is depicted in Fig. 5b.

## Discussion

### Main findings

In the present analysis, pretreated patients with high-grade gliomas of the right hemisphere had significantly lower self-reported HRQoL scores in the domains of physical, role, emotional, and social functioning compared to patients with left hemispheric tumors. These HRQoL impairments were mainly associated with the involvement of the right-sided anterior and posterior limbs of the internal capsule, corona radiata, and striae terminales of the fornix by T2/FLAIR lesions and reduced connectivity of most of the nodes of the right somato-motor and ventral attention networks. In contrast, patients with left-sided gliomas were more prone to aphasia and suffered from communication deficits to a greater extent, which were more strongly associated with the involvement of the left corona radiata and striae terminales of the fornix.

### Health-related quality of life in high-grade glioma patients

While most clinical studies on malignant glioma focus on oncologic outcomes, such as overall and progression-free survival, few reports deal with the detailed evolution of HRQoL subdomains in the course of the disease [5, 27–32]. In newly diagnosed high-grade gliomas, Klein et al. [29] observed a significant decrease in physical and social functioning, vitality, general health perception, and general mental health and increased role limitations compared to a group of healthy subjects. In 2017, Gately et al. [5] reviewed the evidence on HRQoL outcomes in long-term survivors of glioblastoma [27, 31, 33]. In a study that applied the modern EORTC-QLQ C30 instrument, the most noticeable reductions of HRQoL were observed in the domains of role and physical functioning, while the mean global health status was largely preserved [31]. Furthermore, Flechl et al. [27] reported that social functioning and cognition were mostly impaired, in contrast to the global health score. These results and the present study suggest that apart from global measures of HRQoL, specific domains, including physical, role, and social functioning, may be predominantly disturbed in glioma patients.

### Interaction between glioma laterality and health-related quality of life

Few reports have attempted to determine the possible interaction between decreased HRQoL and lesion location or other tumor-related properties. In newly diagnosed high-grade glioma patients, Klein et al. [29] found that although tumor lateralization was significantly correlated self-reported difficulties in specific neurocognitive functions such as reasoning, problem-solving, reaction time, forgetfulness, and concentration, two higher-order HRQoL component scores (physical component scale, mental component scale) did not depend on the side of the affected hemisphere. Several other studies have also not shown any prominent differences in overall HRQoL between left- and right-sided brain tumors in the preoperative or early postoperative phase [34–36]. However, as expected, patients with left-sided tumors reported suffering more often from disturbances in verbal fluency, verbal learning, memory problems, and communications deficits, while tumors in the right hemisphere caused greater difficulties in process skills when performing everyday tasks [35, 36]. These results suggest that measures of global health should be interpreted with caution, as this scale may be insensitive to detect changes over time or between groups.

In contrast, a group from Finland investigated 101 newly diagnosed benign and malignant (41%) brain tumors and found close relations between the right hemispheric tumor load and several HRQoL measures. In detail, the right-sided tumors were associated with a larger overall HRQoL reduction than the left-sided tumors for all domains except speech, and with significantly higher scores for social isolation and emotional deficits. Also, overall HRQoL related to the histological grade and location of the tumor in only the right hemisphere but not in the left [37].

### Neural correlates of health-related quality of life in glioma patients

While several studies have been performed to elucidate the underlying causes of neurocognitive deterioration in glioma patients by modern neuroimaging methods, including voxel-wise lesion-symptom mapping, fMRI, and tractography [38–43], these approaches have rarely been applied to self-reported HRQoL data [44]. It is reasonable to assume that apart from the presence of distracting symptoms, quality of life depends on various functional abilities, such as perception of sensory and emotional stimuli, attention, planning and execution of complex actions, language, speech, and memory. However, in contrast to neurocognitive testing where more specialized functions are measured in isolation, the self-assessment of these abilities by a questionnaire may be more difficult, partly because patients with neurocognitive deficits may have reduced self-awareness, and therefore cannot reliable estimate and report on their functioning and wellbeing.

Hemispheric specialization has recently been reviewed from theoretical [45], clinical [46], and neurosurgical perspectives [47], pointing to sets of tasks predominantly processed in the left (communicative speech and language, mathematical and logical reasoning, encoding episodic memory) or right hemisphere (attentional reorientation, emotional processing, nonverbal semantic processing, spatial cognition, mentalizing, affective prosody, face processing, episodic memory retrieval). In line with these findings, the present results suggest that the tracts and nodes in the right hemisphere are important for daily functioning, which is probably the prerequisite for a good subjective quality of life.

Concerning the type of lesions, we found that the involvement of right-sided white-matter tracts by T2/FLAIR hyperintensities was most strongly correlated with several functional HRQoL domains. Whether these lesions were caused by radiation-induced astrogliosis or by perifocal edema in the presence of a recurrent tumor is challenging to decide. However, in a recent analysis, we found that both types of lesions reduce the fiber density in the underlying white matter by approximately 50% [48].

### Limitations of the Study

The main limitation of this study is the substantial variability in age, time since therapy initiation, therapy intensity, and anticonvulsive medication of the patients. Such, some patients may have had time to adapt to their situation while others were still troubling with the daily demand of life and the presence of symptoms. In addition, the items in the EORTC questionnaires were sometimes difficult for tumor patients to understand, which may have introduced some reporting bias into the data.

## Conclusion

In pretreated high-grade glioma patients, HRQoL measures for most domains except communication abilities were less favorable in right hemisphere compared to left hemisphere tumors. These impairments were mainly associated with T2/FLAIR lesions involving white-matter tracts and functional node connectivity of the right hemisphere. Thus, sparing the right hemisphere from treatment-induced tissue damage should be considered to improve HRQoL in glioma patients.

## Supplementary Information

Below is the link to the electronic supplementary material.

Supplementary file1 (PDF 616 KB)

## Data Availability

The datasets generated during and/or analyzed during the current study are available from the corresponding author on reasonable request.
