# Supplementary material for: The impact of brain lesions on health-related quality of life in patients with WHO CNS grade 3 or 4 glioma: a lesion-function and resting-state fMRI analysis
Source: J Neurooncol. 2023 Feb 7;161(3):643–54. doi: 10.1007/s11060-023-04254-1 (PMC9992025; doi:10.1007/s11060-023-04254-1)
Supplement: Supplementary file 1 — Supplementary file1 (PDF 616 KB) [file 11060_2023_4254_MOESM1_ESM.pdf]

Supplementary Information

The impact of brain lesions on health-related quality of life in patients with WHO CNS grade 3 or 4 glioma: A lesion-function and resting-state fMRI analysis

J Neuro-Oncology

Alexander Heinzel, Felix M Motthagy, Christian Filss, Gabriele Stoffels, Philipp Lohmann, Michel Friedrich, Nadim J Shah, Svenja Caspers, Carolin Weiss Lucas, Maximilian I Ruge, Norbert Galldiks, Gereon R Fink, Karl-Josef Langen, Martin Kocher

Correspondence:
Prof. Dr. Martin Kocher
Institute of Neuroscience and Medicine, Research Center Juelich, Juelich, Germany
martin.kocher@uk-koeln.de

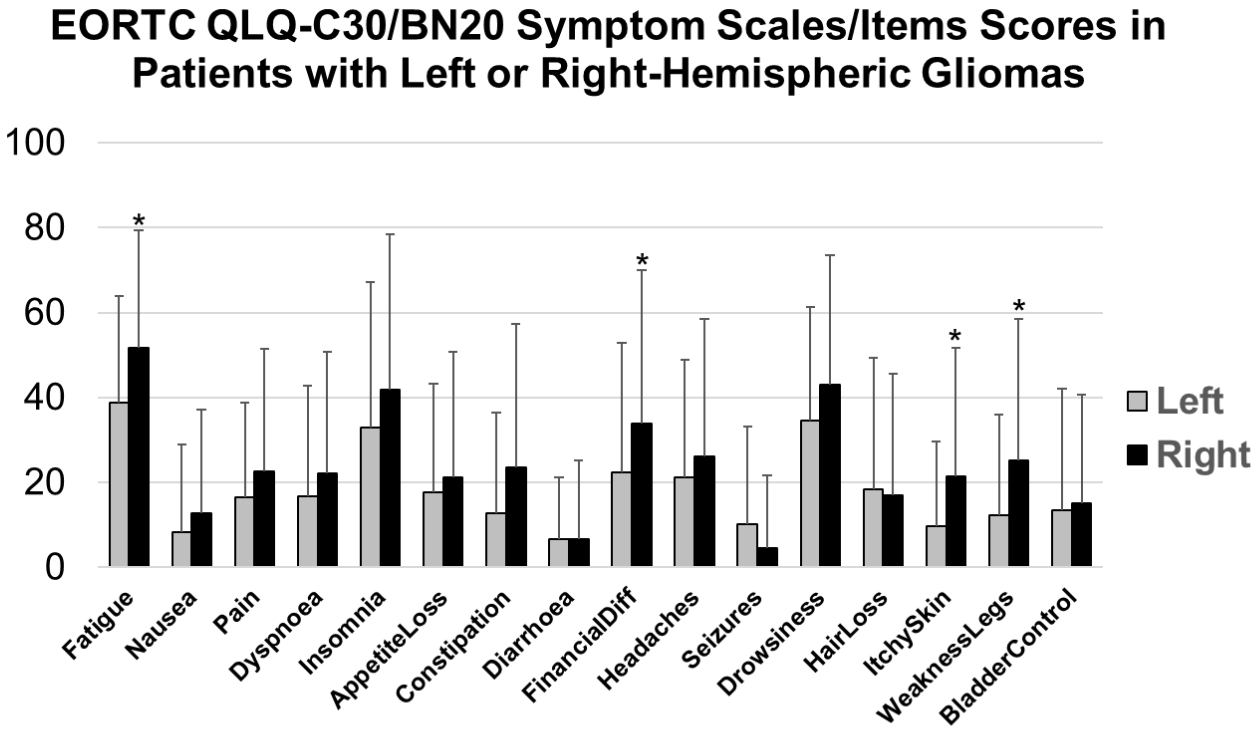

Fig. S1 EORTC QLQ-C30/BN20 symptom scales/items scores in patients with left or right-sided gliomas. *p<0.05, Mann-Whitney U-test. For all scales/items, higher scores indicate higher symptom severity.

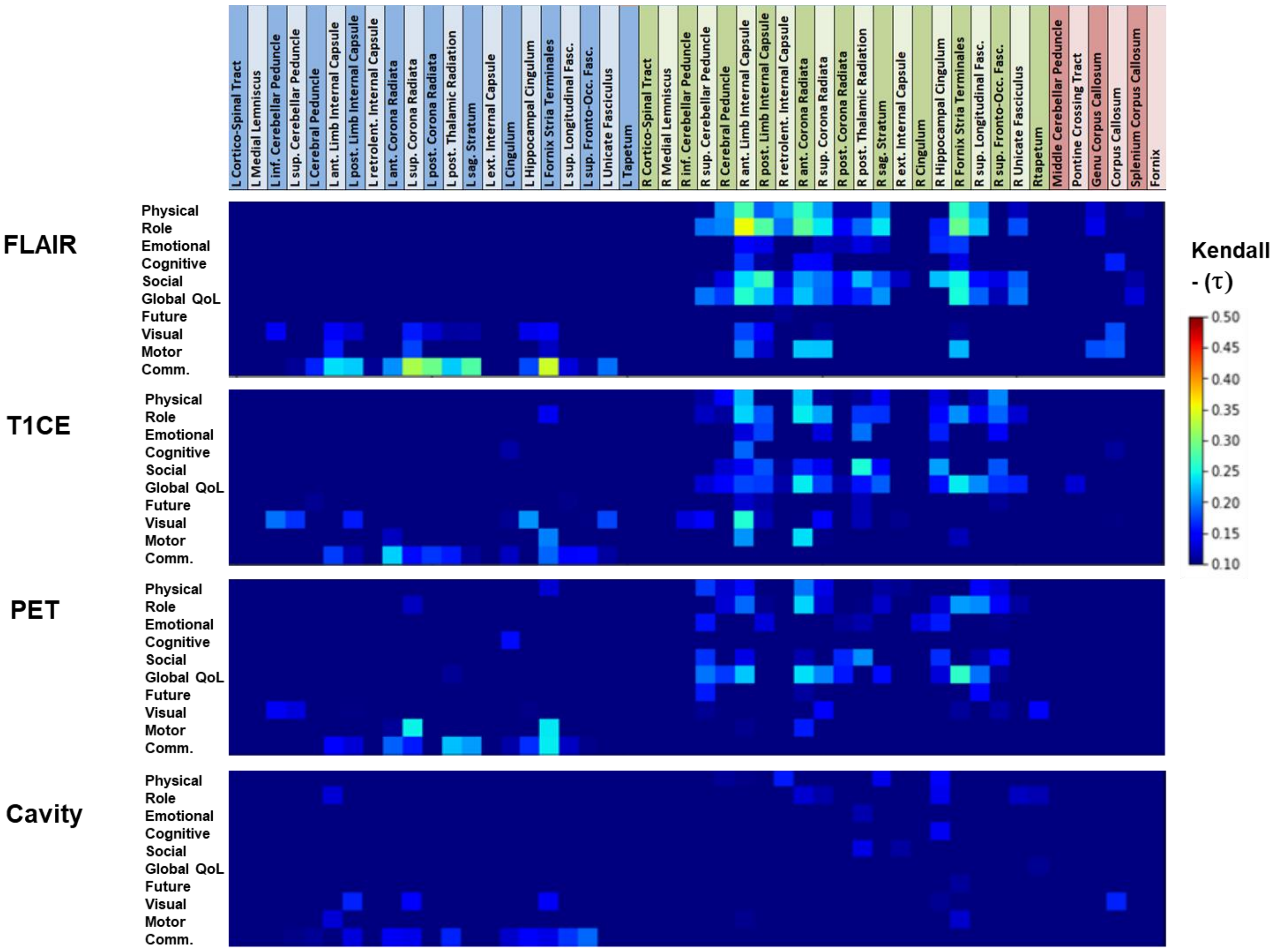

Fig. S2 Heatmap for the negative correlation coefficients (Kendall tau) between partial volumetric affection of white-matter tracts and EORTC HRQoL scores in patients with high-grade gliomas. The top row shows the list of left-sided (blue), right-sided (green), and midline (red) tracts. Physical: physical functioning; Role: role functioning; Cognitive: cognitive functioning; Social: social functioning; Global QoL: global health status/ QoL; Future: future uncertainty; Visual: visual disorder; Motor: motor dysfunction; Comm.: communication deficit

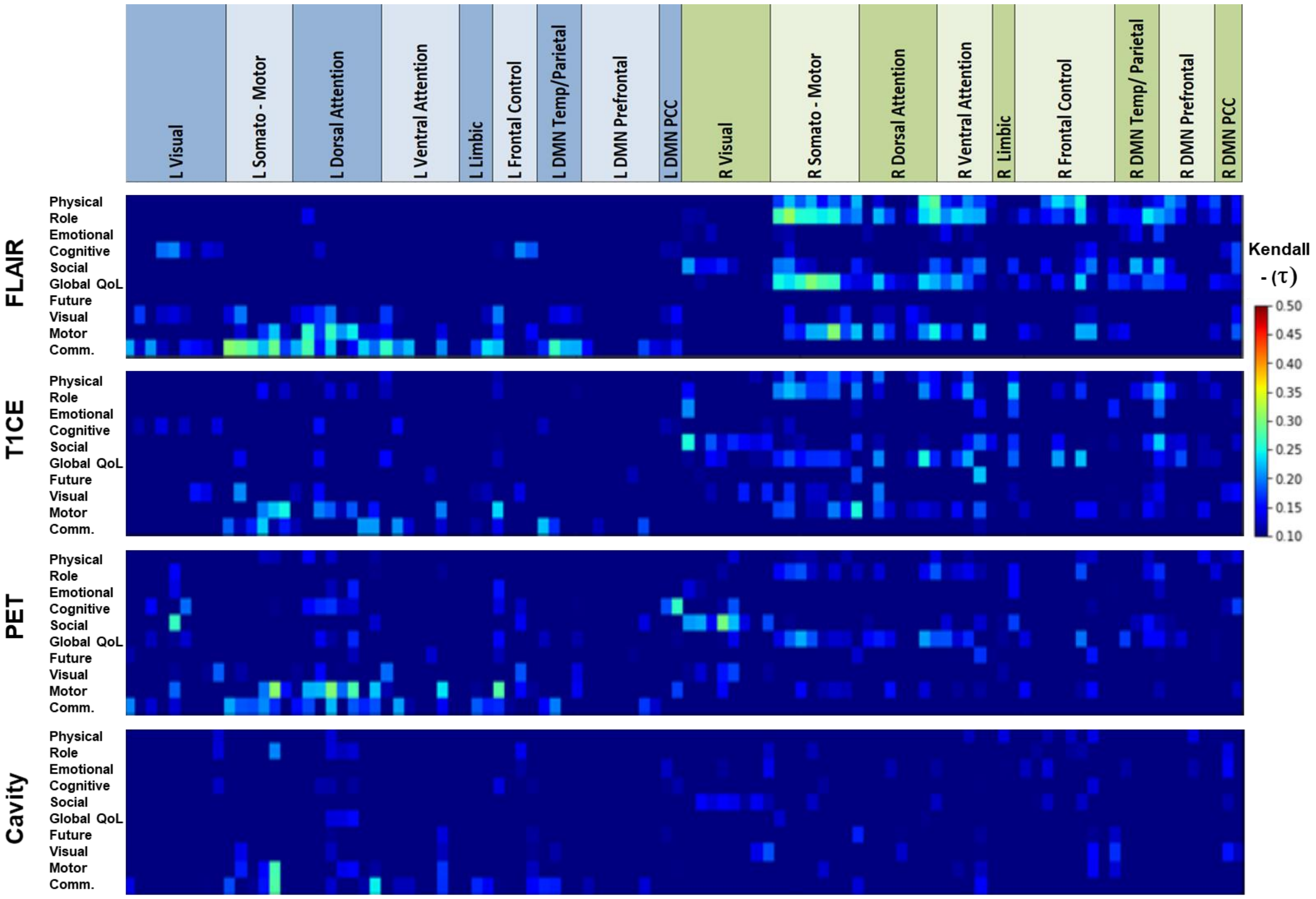

Fig. S3 Heatmap for the negative correlation coefficients (Kendall tau) between volumetric affection of functional cortical areas and EORTC HRQoL scores in patients with high-grade gliomas. The top row indicates membership of the 2x50 nodes to the left-sided (blue) and right-sided (green) parts of 7 resting-state networks. DMN: default mode network; PCC: posterior cingulate cortex. Physical: physical functioning; Role: role functioning; Cognitive: cognitive functioning; Social: social functioning; Global QoL: global health status/ QoL; Future: future uncertainty; Visual: visual disorder; Motor: motor dysfunction; Comm.: communication deficit

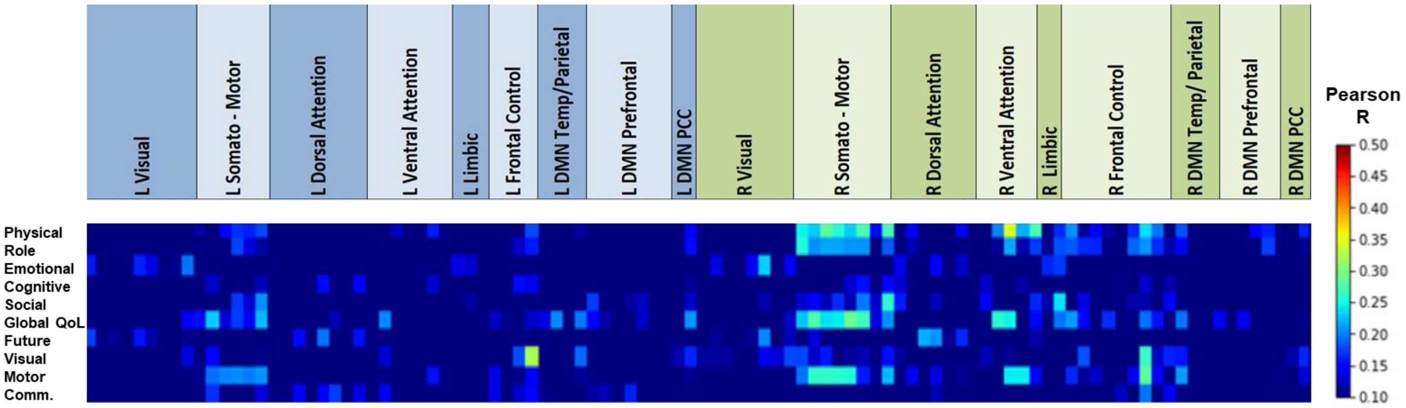

Fig. S4 Heatmap of positive Pearson correlation coefficients for within-network functional connectivity of individual cortical nodes and EORTC HRQoL scores in patients with high-grade gliomas. The top row indicates the membership of the 2x50 nodes to the left-sided (blue) and right-sided (green) parts of 7 resting-state networks. DMN: default mode network; PCC: posterior cingulate cortex. Physical: physical functioning; Role: role functioning; Cognitive: cognitive functioning; Social: social functioning; Global QoL: global health status/ QoL; Future: future uncertainty; Visual: visual disorder; Motor: motor dysfunction; Comm.: communication deficit
